# Role of Intestinal Microbiota in Metabolism of Gastrodin In Vitro and In Vivo

**DOI:** 10.3390/metabo9040069

**Published:** 2019-04-08

**Authors:** Mahesh Raj Nepal, Ki Sun Jeong, Geon Ho Kim, Dong Ho Cha, Mi Jeong Kang, Jin Sung Kim, Ju-Hyun Kim, Tae Cheon Jeong

**Affiliations:** College of Pharmacy, Yeungnam University, 280 Daehak-Ro, Gyeongsan 38541, Korea; maheshnpl10@gmail.com (M.R.N.); balunboy@naver.com (K.S.J.); kgh151300@naver.com (G.H.K.); chadh0309@naver.com (D.H.C.); mjkang@ynu.ac.kr (M.J.K.); whitehall7@naver.com (J.S.K.)

**Keywords:** gastrodin, 4-hydroxybenzyl alcohol, antibiotics, intestinal microbiota, metabolism, LC-MS/MS

## Abstract

Alteration in the number and composition of intestinal microbiota affects the metabolism of several xenobiotics. Gastrodin, isolated from *Gastrodia elata,* is prone to be hydrolyzed by intestinal microbiota. In the present study, the role of intestinal microbiota in gastrodin metabolism was investigated in vitro and in vivo. Gastrodin was incubated in an anaerobic condition with intestinal contents prepared from vehicle- and antibiotics-treated rats and the disappearance of gastrodin and formation of 4-hydroxybenzyl alcohol (4-HBA) was measured by liquid chromatography coupled to mass spectroscopy (LC-MS/MS). The results showed that almost all gastrodin incubated with control intestinal contents was metabolized to its aglycone in time- and concentration-dependent manners. In contrast, much less formation of 4-HBA was detected in intestinal contents from antibiotics-treated rats. Subsequently, in vivo pharmacokinetic study revealed that the antibiotic pretreatment of rats significantly affected the metabolism of gastrodin to 4-HBA. When administered orally, gastrodin was rapidly absorbed rapidly into plasma, metabolized to 4-HBA, and disappeared from the body within six hours. Interestingly, the pharmacokinetic parameters of 4-HBA were changed remarkably in antibiotics-treated rats, compared to control rats. The results clearly indicated that the antibiotics treatment of rats suppressed the ability of intestinal microbiota to metabolize gastrodin to 4-HBA and that, thereby, the pharmacodynamic action was significantly modulated.

## 1. Introduction

In human intestine, trillions of bacteria do not only play major roles in retrieving energy from carbohydrates, proteins, and fats, but also synthesize or metabolize vitamins B and K and bile salts [1]. In recent years, possible roles of intestinal microbiota in xenobiotic metabolism have been extensively studied [2]. From studies, numerous pieces of evidence for the alteration of pharmacological and toxicological effects of xenobiotics by intestinal microbiota have also been provided [1,2]. Now, it is generally accepted that intestinal microbiota can initiate the metabolism of many xenobiotics including drugs by several mechanisms, such as hydrolysis, reduction, acetylation, decarboxylation, and dehydroxylation, by which either toxicity or efficacy of certain xenobiotics are greatly modulated [3].

**Figure 1:**
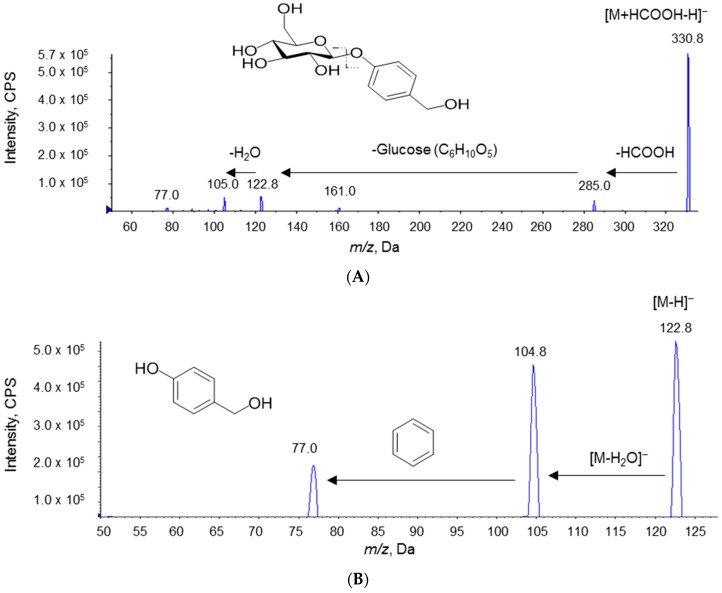
Chemical structures and product ion spectra of gastrodin (**A**) and 4-hydroxybenzyl alcohol (**B**).

Gastrodin (Figure 1A) is the main bioactive constituent isolated from *Gastrodia elata* and *Galeola faberi*, which are widely distributed in Asian countries, including Nepal, India, Korea, Japan, and China [4]. Gastrodin has been used as a nutrient supplement for centuries [5]. Because gastrodin is a glycoside of 4-hydroxylbenzyl alcohol (4-HBA; Figure 1B), it is prone to be hydrolyzed into its aglycone [6]. Both gastrodin and 4-HBA have wide pharmacological effects and have been used in various clinical conditions, such as headache, migraine, and paralysis [4,5]. They were also investigated to have hepatoprotective, anti-oxidant, anti-tumor, memory-enhancing, anti-depressant, and anti-atherosclerotic effects [4,5]. Moreover, the neuroprotective effects of gastrodin and 4-HBA, after having passed the blood–brain barrier, have also been reported [4]. Nevertheless, it was of interest to determine the pharmacologically active forms of the drug when treated in vivo, because glycosidic compounds usually undergo metabolism to their aglycone forms prior to the intestinal absorption [7].

Although the clinical applications of gastrodin and its metabolites, 4-HBA, have been investigated with respect to their pharmacokinetic characteristics [8,9,10], the effects of intestinal microbiota on gastrodin metabolism and its pharmacokinetics have not been extensively studied in vivo and in vitro. Herein, we investigated the roles of intestinal microbiota in gastrodin metabolism and pharmacokinetics of gastrodin by using liquid chromatography coupled to mass spectroscopy (LC-MS/MS). To control the intestinal microbiota, a combination of three antibiotics, which were already proven to be effective in our laboratory, were orally administered to rats prior to gastrodin administration [11]. Then pharmacokinetic parameters of oral gastrodin were compared between vehicle-treated and antibiotics-treated rats. For the in vitro studies, an anaerobic gas-generating pouch system was employed to better mimic the intestinal environment. Depletion of gastrodin and formation of 4-HBA were observed by LC-MS/MS following in vitro incubation of gastrodin with intestinal contents under the anaerobic gas-generating pouch system.

## 2. Results

### 2.1. Method Development and Validation

For the accurate assessment and quantification of gastrodin and 4-HBA, an LC-MS/MS method was developed. As depicted in Figure 1A and 1B, both gastrodin and 4-HBA were ionized at negative ion modes. Gastrodin formed the formic acid adduct [M+HCOOH-H]^−^ at *m/z* 330.8, as reported in previous reports [12,13]. With the loss of formic acid, a precursor ion [M−H]^−^ at *m/z* 285.0 was detected. By the detachment of glucose, a product ion was detected at *m/z* 122.8. Similarly, 4-HBA was ionized as [M−H]^−^ at *m/z* 122.8 and fragmented to the ion at *m/z* 104.8 by the removal of H_2_O. Tolbutamide (IS) was ionized at *m/z* 269.1 and further fragmented to the ion at *m/z* 169.9 (data not shown).

**Figure 2:**
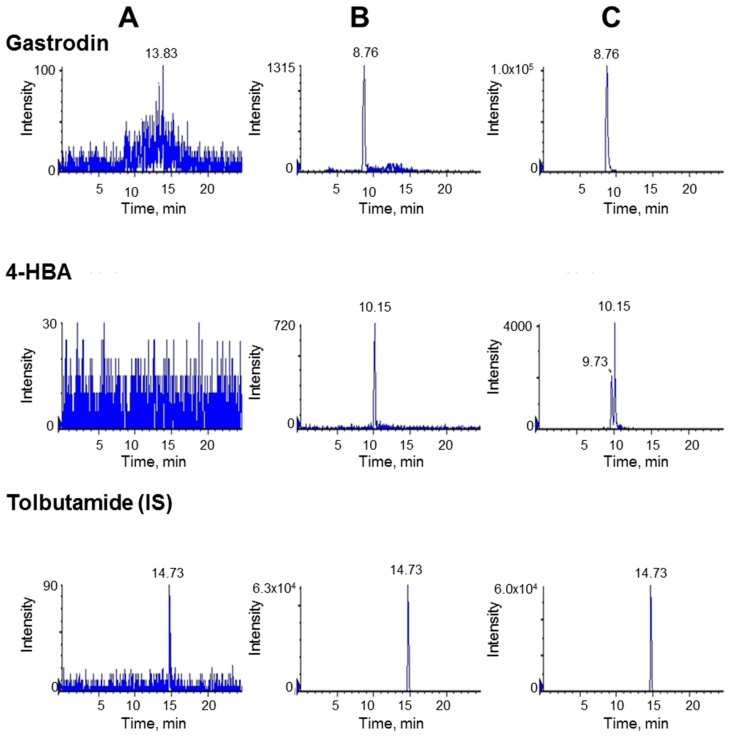
Representative liquid chromatography coupled to mass spectroscopy (LC-MS/MS) chromatograms of gastrodin, 4-HBA, and tolbutamide (IS). Left vertical panel (**A**), blank plasma samples; central vertical panel (**B**), blank plasma sample spiked with analyte at the lower limit of quantification (LLOQ); and right vertical panel (**C**), plasma sample obtained 30 min after oral administration with gastrodin at 40 mg/kg.

Typical chromatograms obtained from blank plasma, a spiked sample with its standard at the lower limit of quantification (LLOQ), and a plasma sample prepared 30 min after in vivo gastrodin administration are presented in Figure 2. No interferences were observed under the developed chromatographic condition. For the in vivo sample, an unknown peak was observed in mass transition of 4-HBA at a retention time of 9.73 min, but this peak was sufficiently separated from 4-HBA and did not affect the quantitation of 4-HBA.

Separate calibration curves were constructed for both gastrodin and 4-HBA during in vivo and in vitro studies. Acceptable linearity was observed at 0.01–20 µg/mL of gastrodin and 0.1–10 µg/mL of 4-HBA. The intra- and inter-day accuracy and precision of gastrodin and 4-HBA are shown in Table 1. The intra-day and inter-day accuracy was less than 101.6% and 106.6% for gastrodin, and 109.7% and 115.0% for 4-HBA, respectively. Similarly, the inter-day and intra-day precision was less than 10.8% and 12.4% for gastrodin and 7.9% and 4.6% for 4-HBA, respectively. Short-term, long-term, and freeze–thaw stability results are shown in Table 2. The results indicate that both gastrodin and 4-HBA were stable with an RSD of <15%. Therefore, it was clear that the developed analytical method could yield accurate, precise, and stable results with good reproducibility.

### 2.2. In Vitro Metabolism of Gastrodin in Vehicle- and Antibiotics-Treated Rats

**Figure 3:**
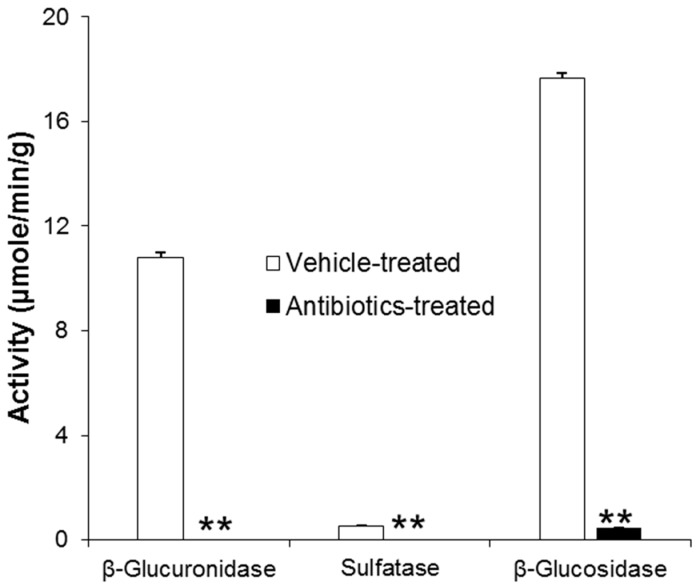
Intestinal microbiota enzyme activities. Asterisks (**) indicate the value significantly different from corresponding controls at *p* < 0.01.

The inhibitory effects of antibiotics treatment on intestinal microbial enzyme activities were confirmed by measuring typical intestinal microbial enzymes, such as β-glucuronidase, sulfatase, and β-glucosidase (Figure 3). The activities of all enzymes in intestinal contents were significantly decreased following oral antibiotics pretreatment for three consecutive days. From the results, it was concluded that the present antibiotics model was appropriate to study the role of intestinal microbiota in pharmacokinetics of gastrodin.

**Figure 4:**
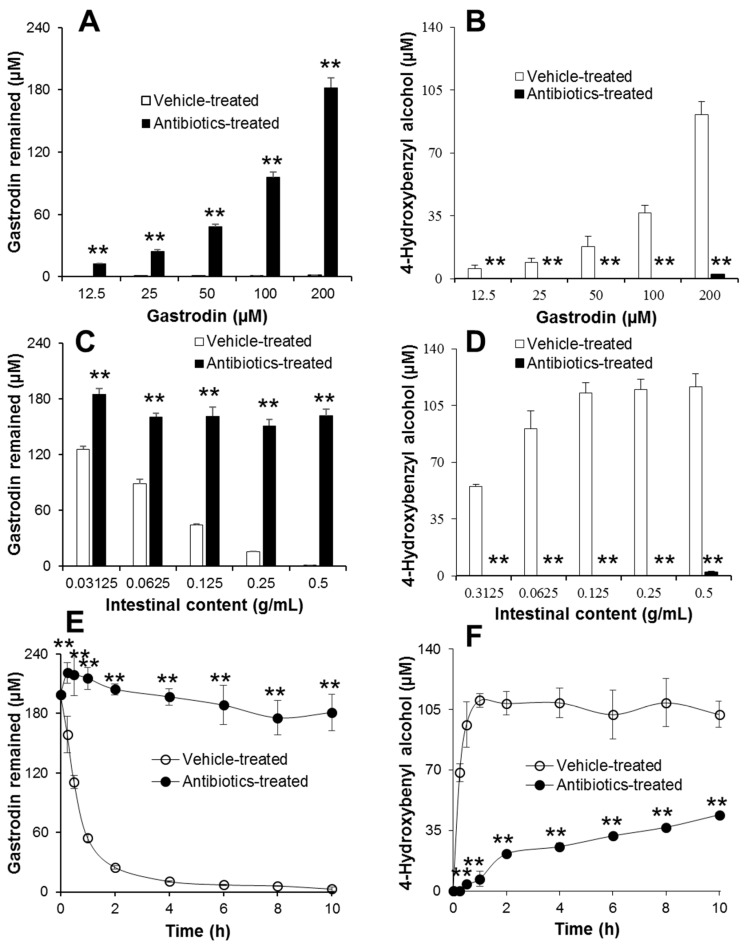
In vitro metabolism of gastrodin by intestinal microbiota. (**A,B**) Concentration-dependent metabolism of gastrodin. Following incubation of various concentrations of gastrodin with 0.5 g/mL intestinal contents prepared from vehicle- and antibiotics-treated rats at 37°C for 4 h, gastrodin and 4-HBA were extracted with methanol containing 10 ng/mL tolbutamide and analyzed by LC-MS/MS. Asterisks (**) indicate the value is significantly different from corresponding control at *p* < 0.01. (**C,D**) Intestinal content-dependent metabolism of gastrodin. Following incubation of 200 µM of gastrodin with various concentrations of intestinal contents prepared from vehicle- and antibiotics-treated rats at 37 °C for 4 h, gastrodin and 4-HBA were extracted with methanol containing 10 ng/mL tolbutamide and analyzed by LC-MS/MS. Asterisks (**) indicate the value is significantly different from corresponding control at *p* < 0.01. (**E,F**) Metabolism of gastrodin over time by intestinal microbiota. Following incubation of 200 µM gastrodin with 0.5 g/mL intestinal contents prepared from vehicle- and antibiotics-treated rats at 37 °C for various time points, gastrodin and 4-HBA were extracted with methanol containing 10 ng/mL tolbutamide and analyzed by LC-MS/MS. Asterisks (**) indicate the value is significantly different from corresponding controls at *p* < 0.01.

The in vitro metabolism of gastrodin was investigated by measuring the amount of gastrodin remaining and of its metabolite, 4-HBA, produced by using LC-MS/MS. The incubation conditions were first optimized by testing various concentrations of gastrodin and various amounts of intestinal contents (Figure 4). Gastrodin was rapidly metabolized to 4-HBA in control groups, whereas less metabolism of gastrodin and formation of 4-HBA were observed in intestinal contents from antibiotics-treated rats (Figure 4A,B). Similarly, the metabolism of gastrodin was also dependent on the amount of intestinal contents used during the incubation with 200 µM gastrodin (Figure 4C,D). It was observed that the higher the amount of intestinal contents used, the higher was the production of 4-HBA. However, no metabolism was observed in groups incubated with intestinal contents from antibiotics-treated rats, indicating that the intestinal microbiota play the major role in the metabolism of gastrodin. Therefore, the highest concentration of gastrodin and the highest amount of intestinal contents were chosen for the time-dependent in vitro metabolism of gastrodin (Figure 4E,F). When 200 µM gastrodin was incubated with 0.5 g/mL intestinal contents from vehicle-treated rats, gastrodin was rapidly metabolized to 4-HBA within 4 h of incubation, whereas most of the gastrodin remained in the reaction mixture with the intestinal contents from antibiotics-treated rats (Figure 4E,F). The formation rate of 4-HBA in the vehicle-treated group was very rapid (up to 2 h of incubation), followed by a steady state thereafter. Meanwhile, the formation of 4-HBA was marginal throughout the sampling period in the antibiotics-treated group. Once again, the results indicate that the intestinal microbiota played a critical role in the metabolism of gastrodin to its aglycone.

### 2.3. Pharmacokinetics of Gastrodin in Rats

**Figure 5:**
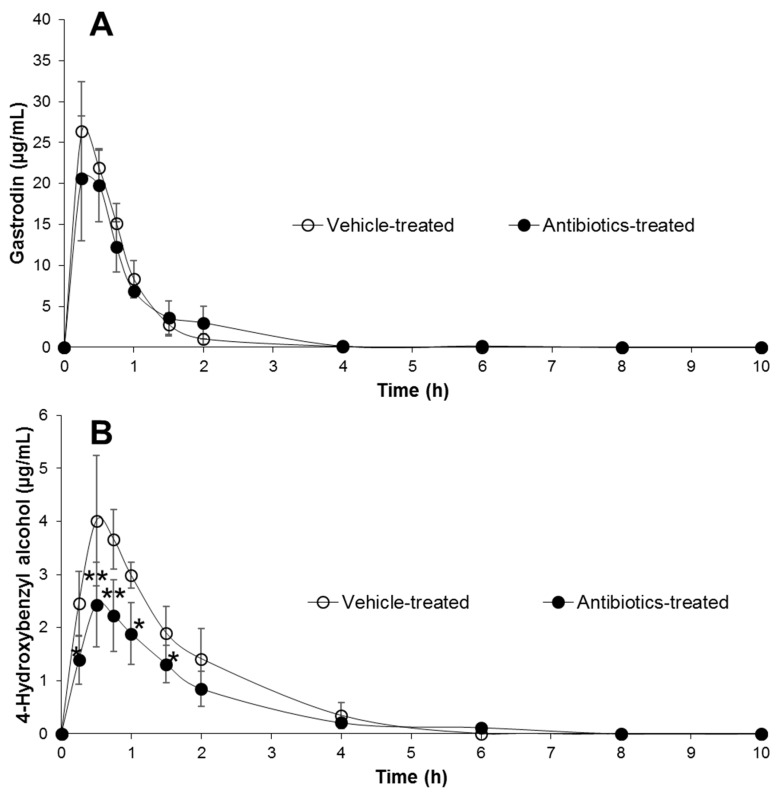
Time course of plasma concentration of gastrodin (**A**) and 4-HBA (**B**) following single oral administration of rats with 40 mg/kg gastrodin in vehicle- and antibiotics-treated rats. Each point represents the mean plasma concentration ± SD of five animals. Asterisks indicate the value significantly different from corresponding controls at either *p* < 0.05 (*) or *p* < 0.01 (**).

After oral administration of gastrodin to rats at 40 mg/kg, the pharmacokinetic profile of gastrodin in antibiotics-treated rats was investigated and compared with that in vehicle-treated rats. The dose of the administered gastrodin was chosen in accordance with a published report [14]. The curves for mean plasma concentration vs. time profile of gastrodin and 4-HBA are shown in Figure 5A,B, respectively, and the pharmacokinetic parameters are summarized in Table 3. After the oral administration of gastrodin, it was rapidly absorbed in its original form with no significant differences between antibiotics-treated and vehicle-treated groups. The C_max_ values of gastrodin in control and antibiotics-treated rats were 26.9 µg/mL and 22.9 µg/mL and the areas under the curve (AUCs) of gastrodin were 21.8 µg·h/mL and 21.9 µg·h/mL, respectively. Although no significant difference was observed in in vivo gastrodin absorption, gastrodin metabolism to 4-HBA in vehicle-treated rats was significantly different from antibiotics-treated rats. By oral pretreatment of rats with antibiotics for 3 days, most of the pharmacokinetic parameters of 4-HBA were significantly affected compared to the control. The C_max_ and AUC of 4-HBA in antibiotic-treated animals were significantly reduced from 4.2 µg/mL and 7.0 µg·h/mL to 2.5 µg/mL and 4.6 µg⋅h/mL, respectively. In addition, the volume of distribution (Vd) and clearance (CL) of 4-HBA were significantly increased in antibiotics-treated groups. The results suggested that the antibiotics pretreatment of rats significantly inhibited or suppressed the intestinal microbiota, resulting in a significant change in the pharmacokinetic profiles of 4-HBA. In addition, it was observed that both gastrodin and 4-HBA were absorbable forms of gastrodin in rats.

## 3. Discussion

Several studies have been conducted to explore the pharmacological activities of gastrodin [6,9,14]. Specifically, both gastrodin and 4-HBA were widely investigated as antioxidants and neuroprotective agents [15,16,17]. The effectiveness of certain medicinal agents, such as gastrodin, was associated with the availability of sugar unit in their structures [18,19]. However, glycosides are more prone to be metabolized by intestinal microbiota than hepatic metabolic enzymes [20]. Therefore, it was critical to investigate the role of intestinal microbiota in gastrodin metabolism to understand its pharmacological actions. Because the fate of gastrodin is largely dependent on the number, composition, and characteristics of intestinal microbiota, it was important to investigate the factors that disturb the intestinal microbiota, which ultimately disturb the metabolism of glycosidic natural products [1,2]. Therefore, in the present study, we investigated the effect of antibiotics on intestinal microbiota and resultant disturbance in gastrodin metabolism in vitro and in vivo.

Several previous studies have reported the use of different antibiotic treatment models for in vivo suppression of intestinal microbiota [21,22]. Various models of antibiotics, such as ampicillin alone or an antibiotic mixture of cefadroxil, oxytetracycline, and erythromycin, were compared with the metabolism of lovastatin to its four metabolites [22]. In addition, in our previous study, the effects of an antibiotic mixture containing oxytetracycline hydrochloride, erythromycin, and cefadroxil were investigated by comparing the number of intestinal bacteria in vehicle- and antibiotics-treated rats [11]. The results showed that the numbers of bacteria in the small intestine, cecum, and large intestine were found to be 5.4, 2.5, and 2.8 times higher in control rats than in antibiotics-treated rats, indicating that a 3-day administration with antibiotics was enough for the effective reduction of bacteria in animals. Moreover, in the present study, three intestinal microbial enzyme activities were measured to confirm the effectiveness of the antibiotic dosing and schedule (Figure 3). The significant decrease in activity of β-glucuronidase, sulfatase, and β-glucosidase indicated that the present antibiotic dosing for three days was sufficient for the effective reduction of intestinal bacteria in antibiotics-treated rats. Thereafter, a pharmacokinetic experiment was conducted 24 h after the last dose of antibiotics. In the in vivo study, less metabolism of gastrodin to 4-HBA was detected in antibiotics-treated rats. Although the measurement of a lower amount of 4-HBA in plasma in antibiotics-administered rats indicated that the pretreatment of oral antibiotics in rats could not absolutely diminish the intestinal microbiota due to the presence of antibiotics-resistant bacteria [11], a significant difference in the pharmacokinetics of 4-HBA in vehicle- and antibiotics-treated rats suggested that the metabolism of gastrodin would solely rely on intestinal microbiota.

Because of the presence of a sugar moiety in the structure of gastrodin, it was believed that gastrodin could not be freely absorbed from the intestine [23]. Despite its hydrophilicity, however, gastrodin was instantaneously metabolized to 4-HBA and both were successively absorbed (Figure 5A,B). In this regard, it was suggested that the P_eff_ value, a criterion to determine the effective intestinal permeability, was very high for gastrodin (>1.0), which was even higher (1.0–2.3) in intestinal segments [24]. Irrespective of gastrodin being a hydrophilic drug, it could be easily absorbed into the body with the help of glucose transporters (GLUT) present in the lumen of the intestine [24]. Because of the rapid absorption of gastrodin via GLUT in rats, no significant difference was achieved in plasma gastrodin level between vehicle- and antibiotics-treated groups in the present study (Figure 5A). Nevertheless, no GLUT receptors were present in extracted intestinal contents. Therefore, gastrodin was rapidly and almost completely metabolized to 4-HBA in control intestinal contents in vitro (Figure 4).

Consistent with the report from a previous study, the major metabolite of gastrodin was identified as 4-HBA, an aglycone [9]. Gastrodin is metabolized to 4-HBA by the enzyme, β-glucosidase, present in human intestinal microbiota, and absorbed through the gastrointestinal tract. Moreover, both gastrodin and 4-HBA were determined to be absorbable forms of gastrodin in the current study, which is consistent with previous findings [4,9]. Some studies pointed towards the formation of some other metabolites including p-hydroxybenzoic acid 4-O-glucoside by oxidation, p-(hydroxymethyl) phenyl glucopyranosiduronic acid by glucuronidation, p-hydroxybenzoic acid and sulfated products of 4-hydroxybenzoic acid, p-hydroxybenzoic acid 4-O-glucoside, and 4-HBA [12]. However, no such metabolites were detected in the present study, except 4-HBA.

Although many glycosides, including baicalin and geniposide, require intestinal microbiota-mediated metabolism to their aglycone forms for their intestinal absorption [11,25], the present study demonstrated that gastrodin also showed good absorption. These results suggest that gastrodin could contribute to its pharmacological actions. In general, however, the possible role of intestinal microbiota in the pharmacodynamic actions of natural products should be considered for better understanding the mode of action because many glycosidic natural products require metabolism to their aglycones in the intestine prior to absorption. Besides sugar-containing drugs or xenobiotics, some sugar-free drugs, such as acetaminophen and metformin, were also reportedly metabolized by intestinal microbiota [26,27]. Therefore, it is generally accepted that the intestinal microbiota can affect the bioavailability and half-life of certain oral xenobiotics including natural products [11]. In this regard, the possible role of intestinal microbiota should be considered in the development of new drug candidates, as well as the classical hepatic metabolism. Particularly, the modulation of intestinal microbiota by antibiotic administration may be clinically relevant. Several side effects, adverse effects, or toxicities of drugs or drug–drug interactions or drug–food interactions will be prevalent if the metabolism of drugs by the intestinal microorganism is not considered. Therefore, effects of metabolism of drugs and other xenobiotics by intestinal microbiota and their effect on pharmacokinetics need to be further explored.

## 4. Materials and Methods

### 4.1. Materials

Gastrodin (>98%) was purchased from Tokyo Chemical Industry. 4-HBA (≥98%), erythromycin, oxytetracycline hydrochloride, cefadroxil, 4-nitrophenyl β-d-glucuronide, 4-nitrophenyl sulfate, and 4-nitrophenyl β-d-glucopyranoside were purchased from Sigma-Aldrich. The anaerobe gas-generating system was purchased from Becton, Dickinson and Company. HPLC-grade methanol was purchased from J. T. Bakers (Central Valley, PA, USA). All other chemicals used were of reagent grades and were used as received.

### 4.2. Animals

Male Sprague-Dawley (SD) rats (250 g ± 10 g) were obtained from Orient Bio (Seoul, Korea). Animals were received at seven weeks of age and randomly placed into two per cage. Animals were acclimatized at least for one week under a controlled temperature of 22 ± 2 °C, a relative humidity of 50 ± 10%, and air changes of 10–20 times per h. Light was maintained at 150–300 Lux in a 12 h light and dark cycle. This study was performed in accordance with the principles of Institutional Animal Care and the Use Committee of Yeungnam University (approval no., 2014-008; approval date, March 10th, 2014). All animals were given access all the time to pure water and diet, consisting of carbohydrate, 44.2%; protein, 18.6%; fat, 6.2%; crude fiber, 3.5%; neutral detergent fiber, 14.7%; ash 5.3%; and minerals, amino acids, vitamins, and fatty acids, 7.5%.

### 4.3. Animal Treatment

Animals were categorized into two groups as vehicle-treated (n = 5) and antibiotics-treated (n = 5). A mixture of cefadroxil (100 mg/kg), erythromycin (300 mg/kg), and oxytetracycline hydrochloride (300 mg/kg) in saline at 10 ml/kg was orally administered to rats in the antibiotics-treated group and saline in the vehicle-treated group once a day for three consecutive days [11]. Twenty-four hours after the last dose of antibiotic administration, animals were sacrificed, and the intestinal contents were collected separately to determine metabolic capacity of intestinal microbiota. Intestinal contents were suspended in two volumes of the weight of intestinal contents in potassium phosphate buffer, pH 7.4, and homogenized for 2 min. The intestinal content was centrifuged at 500 *g* for 10 min at 4 °C [28]. Supernatants were collected and stored at −70 °C until use. Enzyme activities of β-d-glucuronidase, sulfatase, and β-d-glucosidase in extracted intestinal contents were determined. The schedule for the antibiotic pre-treatment was the same for the pharmacokinetic study.

### 4.4. Analytical Conditions

An HPLC (Agilent 1260 system, Santa Clara, CA, USA) coupled with mass spectrometer (API-4000, AB Sciex, Framingham, MA, USA) was used for the analysis of gastrodin and 4-HBA. Analytes were separated with Atlantis C_18_ column (2.1 × 150 mm, 3 µm, Agilent). The mobile phase consisted of 0.1% formic acid in distilled water (A) and methanol (B). The gradient conditions for the analysis were as follows: mobile phase B was started at 2%, increased to 30% within 3 min, increased to 75% in the next 2 min, and again increased to 99% in another 1.5 min. The mobile phase B was on hold at 99% for another 2.5 min, returned to 2% in the next 1.5 min, and was on hold for 13.5 min to equilibrate the pressure in the column. The temperature of column and auto-sampler during analysis was maintained at 45 °C and 4 °C, respectively. The flow rate was maintained at 0.2 mL/min and the injection volume was 5.0 µl. Gastrodin, 4-HBA, and tolbutamide were detected at negative ion mode, and the mass transitions obtained were *m/z* 330.8 → 122.8 for gastrodin, *m/z* 122.8 → 104.8 for 4-HBA, and *m/z* 269.1 → 169.9 for tolbutamide. Multiple reaction-monitoring mode (MRM) was used for the quantitative detection of parent as well as its metabolite ions, using the ratio of area under the curve (AUC) of analyte to IS peaks. The DP, CE, and CXP values were optimized at −45, −22, and −7 for gastrodin; −45, −16, and −5 for 4-HBA; and −60, −34, and −5 for IS, respectively.

### 4.5. Analytical Validation

The method was validated according to the FDA guideline [29]. Accuracy and precision expressed as coefficient of variance (CV%) were calculated based on the analysis of spiked QC samples of four different concentrations of gastrodin and 4-HBA. QC samples of gastrodin at 0.01–20 µg/mL and 4-HBA at 0.1–10 µg/mL were analyzed by LC-MS/MS. The stability of gastrodin was evaluated using three QC samples intended to store at ambient conditions. Short-term stability studies were conducted following the storage of QC samples at room temperature (25 °C) for two days and in the refrigerator (+4 °C) for seven days. Long-term stability was tested following the storage of QC samples at –20 °C for 30 days. The freeze–thaw stability of gastrodin and 4-HBA was also conducted after three freeze and thaw cycles from –20 °C to room temperature. Following the storage of samples at various conditions, the samples were analyzed by LC-MS/MS.

### 4.6. Assays of β-Glucuronidase, Sulfatase, and β-Glucosidase Activities

The enzyme activities were measured based on a previously reported method with some modifications [30]. Briefly, the reaction mixture contained 0.4 mL of either 2.5 mM of 4-nitrophenyl β-d-glucuronide, potassium 4-nitrophenyl sulfate, or 4-nitrophenyl β-d-glucopyranoside in potassium phosphate buffer, pH 7.4, with 0.4 mL of 0.1 M potassium phosphate buffer, pH 7.4, and 0.2 mL of intestinal contents from antibiotics- and vehicle-treated rats. The samples were incubated at 37 °C for 30 min and the reaction was terminated by adding 0.5 mL of 1 N sodium hydroxide. The samples were vortexed and centrifuged at 3,000 *g* for 10 min and the absorbance of resulting supernatants was measured at 405 nm by using UV spectrophotometry (Eppendorf, Germany). Standard calibration curves were prepared with p-nitrophenol for the calculation of individual activities.

### 4.7. In Vitro Metabolism of Gastrodin

For the concentration-dependent metabolism study, 20 µl of various concentrations of gastrodin was incubated with 180 µL of 0.5 g/mL intestinal contents in an anaerobic gas-generating pouch system at 37 °C for 4 h. The exact procedure of the manufacturer’s instructions was followed for the preparation of the anaerobic gas-generating pouch system. Following incubation, 20 µl of incubated sample was mixed with 480 µL of methanol containing 10 ng/mL of tolbutamide (IS). The samples were vortexed and centrifuged at 13,000 *g* for 10 min at 4 °C and 200 µl of supernatants were collected in vials and analyzed by LC-MS/MS. Similarly, for the microbiota-dependent metabolism study, 20 µL of gastrodin at 2 mM was incubated with 180 µl of various concentrations of intestinal contents in an anaerobic gas-generating pouch system at 37 °C for 4 h. Following incubation, 20 µl of incubated samples were mixed with 480 µL of methanol containing 10 ng/mL of tolbutamide. The samples were vortexed and centrifuged at 13,000 *g* for 10 min at 4 °C and 200 µL of supernatants were collected in vials and analyzed by LC-MS/MS. For the time-dependent metabolism study, 20 µl of gastrodin at 2 mM was incubated with 180 µL of intestinal content (0.5 g/mL) in an anaerobic gas-generating pouch system at 37 °C for 0, 0.25, 0.5, 1, 2, 4, 6, 8, and 10 h. Following incubation at individual time points, 20 µl of incubated sample was mixed with 480 µl of methanol containing 10 ng/mL of tolbutamide (IS). The sample was vortexed and centrifuged at 13,000 *g* for 10 min at 4 °C. Then, 200 µl of supernatant was collected into vials and analyzed by LC-MS/MS. Calibration curves of gastrodin and 4-HBA were prepared to determine their concentrations in the incubation mixtures.

### 4.8. Pharmacokinetic Study of Gastrodin

Gastrodin dissolved in saline was orally administered to vehicle- and antibiotic-treated rats at a dose of 40 mg/kg at 24 h after the final dose of antibiotics. Blood samples (150 µL) were collected from subclavian vein of rats in heparinized tubes at 0, 0.25, 0.5, 0.75, 1, 1.5, 2, 4, 6, 8, and 10 h after gastrodin administration. Blood samples were centrifuged at 3,000 *g* for 10 min at 4 °C, and the resulting plasma was pipetted into Eppendorf tubes and stored at −70 °C until use. Twenty microliter of plasma sample was mixed with 60 µL of methanol consisting of 10 ng/mL tolbutamide. Samples were vortex-mixed and centrifuged at 12,000 *g* for 10 min at 4 °C and supernatants were collected in vials and analyzed by LC-MS/MS. Calibration curves were prepared and analyzed accordingly.

### 4.9. Pharmacokinetic Parameters and Statistical Analysis

Pharmacokinetic parameters of gastrodin and its metabolite were obtained from time-vs.-plasma concentration curves by plotting area ratios of analyte to IS, as previously reported [31]. Briefly, pharmacokinetic parameters were calculated by non-compartmental analysis of WinNonlin (version 2.1; Scientific Consulting). Several pharmacokinetic parameters, such as maximum plasma concentration (C_max_), time to reach maximum plasma concentration (T_max_), AUC, terminal half-life (T_1/2_), volume of distribution (Vd), and clearance (CL) were obtained. The results were expressed as mean ± SD of five animals, and the statistical significance of the results was analyzed using Student’s *t*-test and expressed as an asterisk (*) at *p* < 0.05 and asterisks (**) at *p* < 0.01.

## Figures and Tables

**Table 1:** Intra-day and inter-day analytical validations of gastrodin and 4-hydroxybenzyl alcohol (4-HBA) in rat plasma.

	Spiked Concentrations (µg/mL)	Intra-day (n = 5)		Inter-day (n = 5)	
		Accuracy %	CV %	Accuracy %	CV %
Gastrodin	0.01	97.0 ± 10.5	10.8	106.6 ± 13.2	12.4
	0.1	94.3 ± 4.5	4.8	93.4 ± 6.1	6.6
	1	101.6 ± 4.8	4.7	98.5 ± 3.8	3.9
	20	93.5 ± 5.8	6.2	94.6 ± 3.1	3.3
4-HBA	0.1	106.0 ± 7.8	7.4	115.0 ± 2.8	2.5
	1	102.7 ± 1.5	1.5	99.0 ± 4.2	4.3
	5	105.0 ± 8.3	7.9	94.1 ± 4.4	4.6
	10	109.7 ± 8.4	7.6	112.2 ± 3.3	3.0

**Table 2:** Stability of gastrodin and 4-HBA in rat plasma stored under ambient conditions.

	Measured Concentrations (% of control)					
	Gastrodin (µg/mL)			4-HBA (µg/mL)		
	0.01	1	20	0.1	1	10
Short-term at 25 °C	100.2 ± 4.0	102 ± 0.0	101 ± 2.8	111 ± 5.7	101 ± 0.0	105 ± 5.7
Short-term at +4 °C	94.5 ± 4.7	102.5 ± 3.5	98.5 ± 1.4	111 ± 7.1	103 ± 1.4	112 ± 8.5
Long-term at −20 °C	102.2 ± 6.8	99.7 ± 0.5	100 ± 1.4	110.5 ± 3.5	102 ± 0.0	102 ± 0.0
Freeze−thaw (−20 °C to 25 °C)	93.8 ± 2.1	99.7 ± 0.5	98.3 ± 1.1	102.5 ± 0.7	97.3 ± 2.7	100.2 ± 12.4

Short-term stability tests were conducted following the storage of QC samples at 25 °C and 4 °C for 2 and 7 days, respectively. The long-term stability test was conducted following the storage of QC samples in freezer (−20 °C) for 30 days. Three freeze−thaw cycles were tested for freeze−thaw stability tests from −20 °C to room temperature. Each value represents the mean percent of either gastrodin or 4-HBA ± S.D. of triplicate tested samples.

**Table 3:** Pharmacokinetic parameters of gastrodin and 4-HBA.

Parameters	Gastrodin		4-HBA	
	Vehicle-treated	Antibiotics-treated	Vehicle-treated	Antibiotics-treated
T_max_ (h)	0.3 ± 0.1	0.3 ± 0.1	0.7 ± 0.2	0.6 ± 0.1
C_max_ (µg/mL)	26.9 ± 5.3	22.9 ± 5.5	4.2 ± 1.0	2.5 ± 0.8*
t_1/2_ (h)	0.6 ± 0.0	0.6 ± 0.2	0.9 ± 0.3	1.1 ± 0.3
AUC (µg·h/mL)	21.8 ± 2.3	21.9 ± 1.4	7 ± 1.4	4.6 ± 1.0*
Vd (L/kg)	1.1 ± 0.2	1.6 ± 0.4	7.4 ± 1.0	14.8 ± 6.6*
CL (L/h/kg)	1.9 ± 0.2	1.8 ± 0.1	5.8 ± 1.4	9.0 ± 2.0*

Vehicle- and antibiotics-pretreated rats were administered orally with gastrodin at 40 mg/kg. Each value represents the mean ± S.D. of five animals. An asterisk (*) indicates the value is significantly different from the vehicle-treated control at *p* < 0.05.
